# Comparison of four MRI diffusion models to differentiate benign from metastatic retropharyngeal lymph nodes

**DOI:** 10.1186/s41747-025-00590-1

**Published:** 2025-05-13

**Authors:** Jun Liu, Li Hua, Fei Wang, Ming Chen, Yinan Sun, Zhi Hu, Luqing Shu, Andong He, Mengxiao Liu, Qing Yang, Juan Zhu, Yinfeng Qian

**Affiliations:** 1 Department of Radiology, The First Affiliated Hospital of Anhui Medical University, Hefei, China; 2 Department of Medical Imaging, Anqing Medical Center of Anhui Medical University, Anqing, China; 3 Department of Laboratory Medicine, Anqing Medical Center of Anhui Medical University, Anqing, China; 4 MR Search & Marketing Department, Siemens Healthineers Co., Ltd., Shanghai, China; 5 Department of Radiology, The First Hospital of Lanzhou University, Lanzhou, China

**Keywords:** Diffusion magnetic resonance imaging, Lymph nodes, Magnetic resonance imaging, Neoplasm metastasis, Retropharyngeal space

## Abstract

**Background:**

Conventional magnetic resonance diffusion-weighted imaging (DWI) and morphological features have limitations in distinguishing benign from metastatic retropharyngeal lymph nodes (RLNs). We aimed to compare the value of continuous-time random walk (CTRW), fractional-order calculus (FROC), stretched-exponential model (SEM), and conventional DWI, in combination with morphological features, for differentiating between the two groups.

**Methods:**

Fifty-nine patients with 68 RLNs (23 benign and 45 metastatic) were enrolled. All patients underwent DWI with 12 *b*-values. Diffusion data were reconstructed using conventional DWI, SEM, FROC, and CTRW models, yielding nine parameters: apparent diffusion coefficient (ADC), distributed diffusion coefficient (DDC)_SEM_, α_SEM_, D_FROC_, β_FROC_, μ_FROC_, D_CTRW_, α_CTRW_, and β_CTRW_. Diffusion parameters and morphological features were compared using Mann–Whitney *U*, independent sample *t*, or χ^2^ tests. Logistic regression analysis was performed to identify the best diffusion indicator for classification and to develop a multiparameter model combining morphological features. Area under the receiver operating curve (AUC) and DeLong tests were used.

**Results:**

Significant differences in diffusion parameters were found between benign and metastatic RLNs, except for α_CTRW_ (*p* ≤ 0.022). Benign RLNs exhibited higher ADC, DDC_SEM_, D_FROC_, and D_CTRW_, while metastatic RLNs had higher α_SEM_, β_FROC_, μ_FROC_, and β_CTRW_. Multivariate logistic regression analysis identified β_CTRW_ as the optimal single diffusion indicator (AUC = 0.913). The combined model of β_CTRW_ with morphological features further improved diagnostic performance and yielded an AUC of 0.948.

**Conclusion:**

β_CTRW_ is an effective noninvasive biomarker for distinguishing between benign and metastatic RLNs. Thus, combining β_CTRW_ with morphological features enhances diagnostic efficiency.

**Relevance statement:**

This study demonstrates that β_CTRW_, derived from the continuous-time random walk diffusion model, when integrated with morphological features, offers a reliable, noninvasive diagnostic approach for differentiating between benign and metastatic retropharyngeal lymph nodes.

**Key Points:**

- Non-Gaussian diffusion metrics outperformed conventional DWI.
- β_CTRW_ was the best indicator for distinguishing benign from metastatic lymph nodes.
- Combining β_CTRW_ with minimal axial diameter further improved diagnostic efficiency.

**Graphical Abstract:**

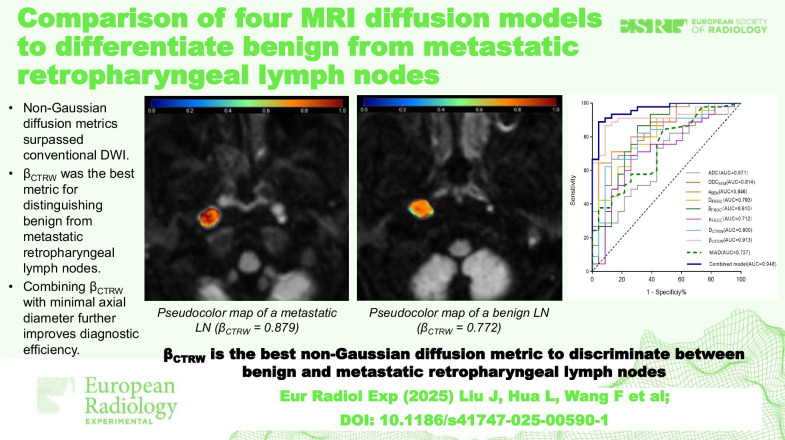

## Background

Nasopharyngeal carcinoma (NPC) is a head and neck cancer associated with the Epstein–Barr virus (EBV), with a high prevalence in southern China and Southeast Asia [1]. It is the most common malignancy leading to metastasis in the retropharyngeal lymph nodes (RLNs), with lymph node metastasis rate exceeding 60% [2–4]. RLNs serve as the first echelon of lymph nodes for NPC metastasis and are independent prognostic indicators [5, 6]. In patients with NPC, cervical lymph nodes < 1.0 cm in diameter are frequently observed, and distinguishing between inflammatory and metastatic lymph nodes is challenging due to overlapping features [7, 8]. Metastatic spread in NPC follows a predictable pattern, with RLN involvement correlating with cervical lymph node metastasis in all neck regions except zone I [2, 8]. Therefore, an accurate assessment of RLN status is critical for evaluating suspected metastatic small lymph nodes in other cervical regions [7–9]. The presence of metastatic RLNs significantly influences treatment choices for patients with NPC, particularly those with stage T1–T3 disease [10]. Specifically, RLN involvement affects the delineation of the clinical target volume of the primary tumor as well as the dosage and extent of radiotherapy [7, 11]. Consequently, precise assessment of RLN metastasis is essential for staging, treatment planning, and prognosis determination.

However, evaluating RLNs is challenging due to their deep anatomical location. The difficulty of surgical clearance and the limited feasibility of biopsy in NPC, which is primarily not surgically treated. Therefore, imaging, particularly magnetic resonance imaging (MRI), plays a crucial role in RLN assessment [1, 11, 12]. RLN metastasis is typically evaluated through clinical and imaging follow-up, with a minimum axial diameter (MiAD) threshold of 5 or 6 mm serving as a diagnostic criterion [13, 14]. However, patients with nasopharyngeal lymphoid hyperplasia and EBV often develop reactive RLN enlargement [15]. This presents a significant overlap between benign reactive hyperplastic and metastatic lymph nodes [16, 17]. Therefore, reliance on MiAD as a diagnostic criterion may result in the misdiagnosis of benign reactive hyperplastic lymph nodes as metastatic.

Diffusion MRI is a widely used noninvasive technique for assessing tissue microcirculation and has demonstrated utility in lymph node evaluation [16, 17], offering insights beyond the conventional morphological characterization of lesions [16–21]. Previous studies have reported low apparent diffusion coefficient (ADC) values in metastatic RLNs [18–20]. However, So et al [21] reported contradictory findings. Furthermore, the existing literature indicates suboptimal diagnostic performance of ADC parameters in differentiating benign from metastatic RLNs, with reported area under the receiver operating characteristic curve (AUC) values consistently below 0.75 [18, 19]. Conventional diffusion-weighted imaging (DWI) models assume a Gaussian distribution of water molecule diffusion, failing to account for tissue heterogeneity [22–38], thereby greatly limiting their applicability [22]. To overcome this limitation, alternative diffusion models, including stretched-exponential (SEM) [22–24], fractional-order calculus (FROC) [25–32], and continuous-time random walk (CTRW) diffusion models [30–38] have been introduced to better characterize non-Gaussian diffusion distribution of water molecule in highly heterogeneous tumor.

The SEM model quantifies intravoxel heterogeneity using the distributed diffusion coefficient (DDC_SEM_) and diffusion heterogeneity coefficient (α_SEM_) [22–24]. SEM can reflect pathological characteristics, the heterogeneity of intravoxel diffusion rates, and the distribution of diffusion effects within multiple water molecule pools in each voxel. Several studies [22–24] have demonstrated that SEM can characterize lesions, such as breast cancer or solid hepatic masses. The FROC model characterizes tissue heterogeneity through three parameters: the derived diffusion coefficient (D_FROC_), spatial fractional-order parameter (β_FROC_), and spatial parameter (μ_FROC_) [25–29]. The FROC model has shown potential in lesion classification, tumor grading, and typing [25–32]. The CTRW model reflects the intratumoral cell density and microstructural heterogeneity through three quantitative parameters: the diffusion constant (D_CTRW_), temporal diffusion parameter (α_CTRW_), and spatial diffusion parameter (β_CTRW_) [33–38]. The CTRW model has been validated in characterizing lesions in the central nervous system, breast, and prostate [31–38]. However, it remains unclear whether the diffusion parameters derived from the CTRW, FROC, and SEM models can effectively differentiate benign from metastatic RLNs. Furthermore, it is uncertain whether these diffusion models outperform ADC values and traditional morphological features in RLN differentiation.

This study aims to determine the value of diffusion parameters derived from conventional DWI, SEM, FROC, and CTRW models, either individually or in combination, in differentiating benign RLNs in plasma EBV-positive patients from metastatic RLNs in NPC patients. Furthermore, we seek to identify morphological differences between the two groups of lymph nodes. Additionally, we sought to evaluate the diagnostic efficacy of these diffusion parameters when combined with morphological features, thus improving valuable references for optimizing N staging and outlining radiotherapy targets.

## Methods

### Patient population

This retrospective study, approved by the Medical Ethics Committee of our hospital, included patients who underwent DWI with 12 *b*-values for the nasopharyngeal region between January 2022 and August 2023. Of 80 patients initially considered, 21 were excluded due to the absence of assessable RLNs or lymph nodes with a MiAD < 6.0 mm (*n* = 14), severe DWI artifacts (*n* = 4), or prior chemotherapy or radiotherapy (*n* = 3). The final cohort comprised 59 patients with 68 RLNs (23 benign and 45 metastatic).

The inclusion criteria for benign RLNs were as follows: (1) EBV deoxyribonucleic acid—DNA positivity with MRI evaluation for nasopharyngeal soft tissue thickening and a final pathological diagnosis of lymphoid hyperplasia; (2) absence of NPC or other head and neck tumors confirmed by MRI and endoscopy, with at least 1-year follow-up; (3) MiAD ≥ 6.0 mm; (4) no previous history of autoimmune diseases or malignancies.

Metastatic RLNs were defined as follows: (1) pathologically confirmed NPC with RLN coverage on nasopharyngeal DWI; (2) presence of at least one lymph node with a MiAD ≥ 6.0 mm, necrosis, or extranodal extension; (3) lymph node resolution after at least 1-year follow-up after NPC treatment.

**Fig. 1:**
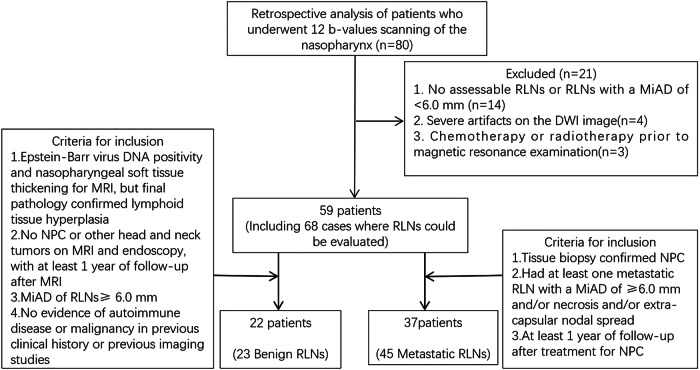
Flow chart of RLNs selection in this study. MiAD, Minimal axial diameter; NPC, Nasopharyngeal carcinoma; RLNs, Retropharyngeal lymph nodes

The inclusion criteria for benign and metastatic RLNs were based on guidelines from previous studies [20, 21]. The RLNs selection process is shown in Fig. 1.

### MRI acquisition

MRI scans were performed using a 3.0-T whole-body system (MAGNETOM Vida, Siemens Healthcare, Erlangen, Germany) with a 20-channel head and neck phased array coil. The scanning sequence protocol included the following:

1. Coronal T2-weighted imaging with fat saturation: repetition time (TR) = 4,330 ms; echo time = 86 ms; field of view = 25 × 25 cm; slice thickness = 4 mm; slice gap = 1 mm; number of excitations = 1;
2. Axial T1-weighted imaging: repetition time = 463 ms; echo time = 6.5 ms; field of view = 20 × 20 cm; slice thickness = 4 mm; slice gap = 1 mm; number of excitations = 3;
3. Axial T2-weighted imaging with fat saturation: TR = 4,330 ms; TE = 86 ms; FOV = 20 × 20 cm; slice thickness = 4 mm; slice gap = 1 mm; number of excitations = 2;
4. DWI sequence using “Readout Segmentation of Long Variable Echo-trains”—RESOLVE, with 12 *b*-values (0, 10, 20, 50, 100, 200, 400, 800, 1,000, 1,500, 2,000, and 3,000 s/mm²), with a single excitation for each *b*-value. The specific parameters were field of view = 22 × 22 cm; repetition time = 3,800 ms; echo time = 70 ms; slice thickness = 4 mm; slice gap factor = 20%; readout segments = 5; number of slices = 22; simultaneous multi-slice = 2; diffusion mode = 3-scan trace; scanning time is approximately 7 min;
5. Finally, contrast-enhanced T1-weighted imaging scans were performed in axial, coronal, and sagittal planes with an intravenous injection of gadodiamide (Omniscan, GE Healthcare Ireland Ltd, Cork, Ireland) at a flow rate of 2.5 mL/s (total dose of 0.1 mmol/kg) via the median cubital vein.

### Image analysis

Diffusion parameters from conventional DWI, SEM, FROC, and CTRW were processed using Body DiffusionLab (BoDiLab, Chengdu ZhongYing Medical Technology Co., Ltd, Chengdu, China) software on a workstation. The final pseudocolor map was generated with ITK-SNAP software http://www.itksnap.org/pmwiki/pmwiki.php.

For conventional DWI, ADC was generated by fitting a single-exponential model using Eq. 1:

1 $$\frac{S(b)}{S(0)}=\exp (-b\cdot {ADC})$$

where *S*(b) represents signal intensity and *S*(0) is the signal intensity without diffusion weighting. The diffusion weighting factor b determines the degree of diffusion weighting in the signal intensity.

The SEM model is fitted using Eq. 2:

2 $$\frac{S(b)}{S(0)}=\exp [-(b\cdot {DDC})^{a}]$$

The distributed diffusion coefficient (DDC) is the diffusion coefficient of the stretched-exponential model, α is the heterogeneity index (dimensionless), and *b* is a complex factor related to diffusion gradient strength and diffusion time.

The FROC model is represented by Eq. 3:

3 $$\frac{S(b)}{S(0)}=\exp \left[-D{\mu }^{2(\beta -1)}{(\gamma {G}_{d\delta })}^{2\beta }\left(\Delta -\frac{2\beta -1}{2\beta +1}\delta \right)\right]$$

where *G*_d_ is the diffusion gradient amplitude, Δ is the gradient interval, and δ is the diffusion gradient pulse width. The parameter β is the intravoxel diffusion heterogeneity parameter, and μ is the spatial diffusion parameter used to retain the nominal units of *D* in mm^2^/s. The diffusion images were fitted to the FROC model using the Levenberg–Marquardt nonlinear fitting algorithm [39]. During the fitting process, lower *b*-values (≤ 1,000 s/mm²) were used to estimate *D* with a monoexponential model. β and μ were derived from image fitting using all *b*-values after *D* was determined.

The CTRW model is fitted using Eq. 4:

4 $$\frac{S(b)}{S(0)}={E}_{\alpha }[{(-{bD})}^{\beta }]$$

where *D* is the anomalous diffusion coefficient, estimated by nonlinear fitting of diffusion images with *b*-values ≤ 1,000 s/mm². α and β are parameters related to temporal and spatial diffusion heterogeneity, respectively, and are determined simultaneously from all diffusion-weighted images.

All lymph node size, signal homogeneity, lymph node borders and diffusion parameters were independently assessed in a blinded manner by two observers with 10 (J.L.) and 15 years (F.W.) of experience in diagnosing head and neck tumors. Using T1-weighted images as references, the maximum and minimal axial diameter of lymph nodes were measured on T2-weighted with fat saturation imaging and further categorized into three dimensions (≥ 6 mm, < 8 mm, ≥ 8 mm, < 10 mm, and ≥ 10 mm) according to the classification methodology established in a previous study [17]. MiAD was defined as the maximum value in the axial plane of the lymph node perpendicular to the maximum axial diameter. Signal homogeneity is categorized into homogeneous and heterogeneous changes. Lymph node borders were categorized as clear or unclear based on the presence or absence of extraperitoneal spread.

**Fig. 2:**
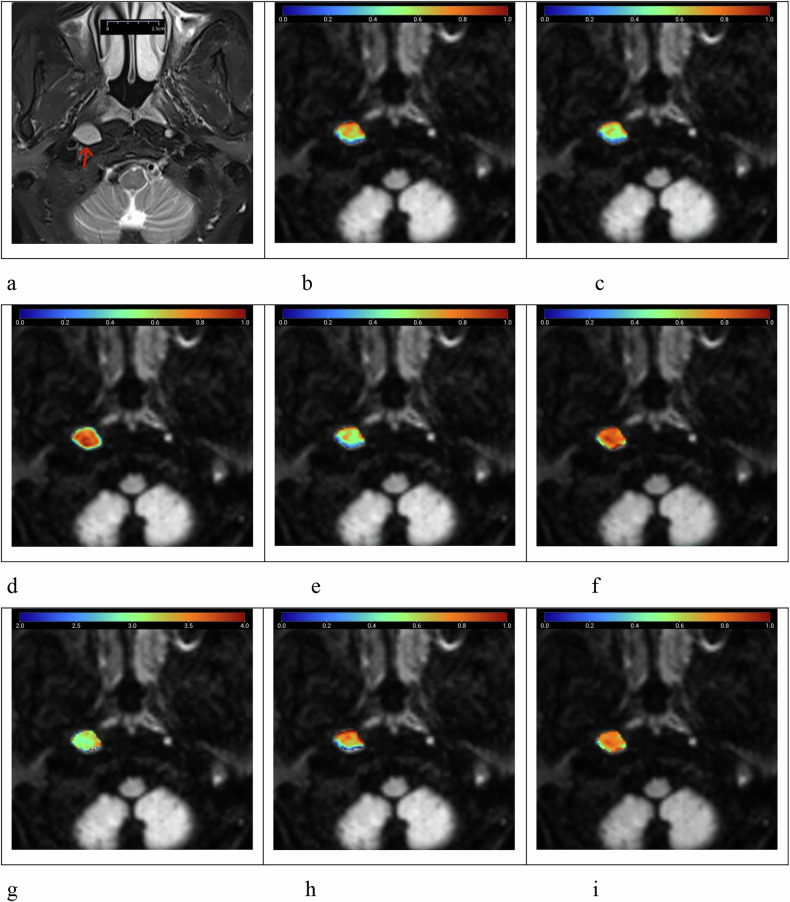
Female patient, aged 45 years, with nasopharyngeal lymphoid hyperplasia and Epstein–Barr virus positivity, presenting with a benign right retropharyngeal lymph node (arrow). **a** T2-weighted imaging with fat saturation shows the lymph node with a maximum axial diameter of 1.58 cm and minimal axial diameter of 1.14 cm, exhibiting a homogeneously slightly high signal. **b**–**i** Pseudocolor maps of the quantitative parameters ADC = 0.678 μm^2^/ms (**b**), DDC_SEM_ = 0.616 μm^2^/ms (**c**), α_SEM_ = 0.740 (**d**), D_FROC_ = 0.597 μm^2^/ms (**e**), β_FROC_ = 0.815 (**f**), μ_FROC_ = 3.289 μm (**g**), D_CTRW_ = 0.721 μm^2^/ms (**h**), and β_CTRW_ = 0.772 (**i**), derived from conventional DWI, SEM, FROC, and CTRW diffusion models. ADC, Apparent diffusion coefficient; CTRW, Continuous-time random walk; DDC, Distributed diffusion coefficient; DWI, Diffusion-weighted imaging; FROC, Fractional-order calculus; SEM, Stretched-exponential model

**Fig. 3:**
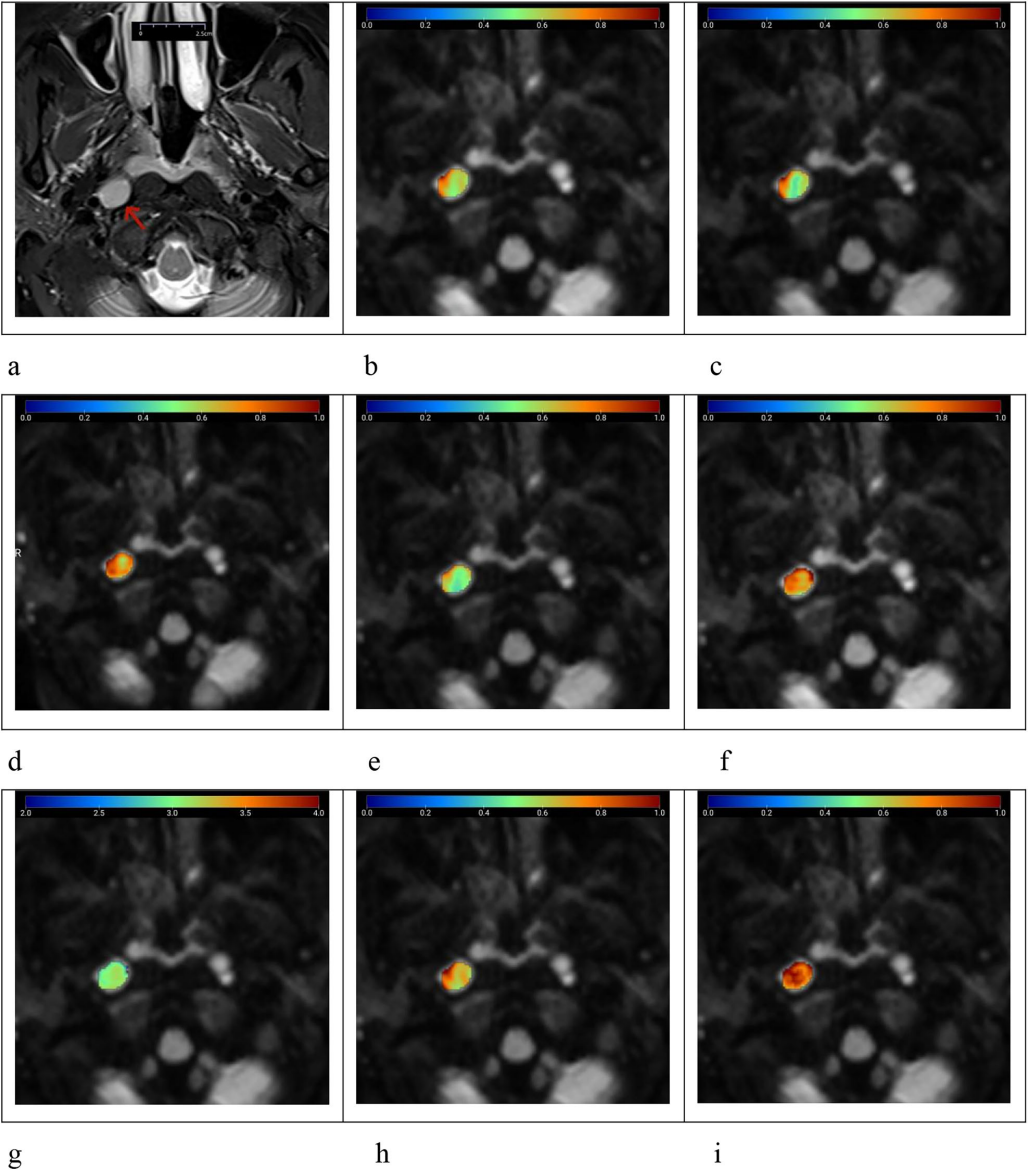
Female patient, aged 51 years, with nasopharyngeal non-keratinizing squamous carcinoma and a metastatic right retropharyngeal lymph node (arrow). **a** T2-weighted imaging with fat saturation shows the lymph node with a homogeneously slightly high signal, a maximum axial diameter of 1.19 cm, and minimal axial diameter of 0.93 cm. **b**–**i** Pseudocolor maps of the quantitative parameters ADC = 0.630 μm^2^/ms (**b**), DDC_SEM_ = 0.612 μm^2^/ms (**c**), α_SEM_ = 0.755 (**d**), D_FROC_ = 0.589 μm^2^/ms (**e**), β_FROC_ = 0.829 (**f**), μ_FROC_ = 3.107 μm (**g**), D_CTRW_ = 0.740 μm^2^/ms (**h**), and β_CTRW_ = 0.879 (**i**), derived from conventional DWI, SEM, FROC, and CTRW diffusion models. ADC, Apparent diffusion coefficient; CTRW, Continuous-time random walk; DDC, Distributed diffusion coefficient; DWI, Diffusion-weighted imaging; FROC, Fractional-order calculus; SEM, Stretched-exponential model

The multi-*b*-value diffusion images were initially imported into the Body DiffusionLab post-processing software, where image registration was performed to ensure accurate alignment. Subsequently, the software applied the mathematical formulas of the CTRW, FROC, SEM, and conventional DWI models to perform pixel-wise fitting. By comparing the signal intensity at each pixel with different *b*-values, the software calculated multiple diffusion-related parameters, which were then presented as pseudocolor maps. Region of interest (ROI) was manually delineated on DWI with a *b*-value of 1,000 s/mm², ensuring the exclusion of necrotic areas, cystic changes, and adjacent anatomical structures. The software then automatically propagated the delineated ROIs across all nine diffusion parameter maps and computed the corresponding values (Figs. 2 and 3). Another independent observer with 20 years of experience in MRI (J.Z.) reviewed the ROIs delineated by the two radiologists and resolved any disputes regarding lymph node borders and signal homogeneity.

The mean values of the two observers’ measurements were used as final parameter values, and the interclass correlation coefficients were calculated to assess interobserver agreement. The same observers repeated the measurements after a 1-month interval, and intragroup correlation coefficients were computed to evaluate intraobserver reliability.

### Statistical analysis

Quantitative parameters were tested for normality. Normally distributed data were represented as mean *±* standard deviation ($\bar{X}$ ± *S*), whereas those with a skewed distribution were represented as median *(P25, P75)*. Data analyses were performed using MedCalc v.22.01 (MedCalc Software Ltd, Ostend, Belgium), SPSS v.23.0 (IBM Corp, Armonk, NY, USA), and GraphPad Prism v.8.0 (GraphPad Software, San Diego, CA, USA). Differences between noncategorical data were assessed using independent sample *t* or Mann–Whitney *U* tests. Categorical data were compared using χ^2^ tests. Significant parameters (*p* < 0.050) identified via binary logistic regression analysis were used to construct a multiparametric diagnostic model and generate the corresponding odds ratios. Receiver operating characteristic curves were generated to assess diagnostic performance, with area under the curves (AUCs) compared using the DeLong test. Interobserver consistency was evaluated using intraclass correlation coefficients with 95% confidence intervals. A two-sided test with *p* < 0.050 was considered statistically significant.

## Results

### Clinical characteristics and grouping of participants

The study analyzed 23 benign and 45 metastatic RLNs from 59 patients. No significant differences were observed in maximum axial diameter, T2 signal homogeneity, sex, age, or borders between benign and metastatic RLNs. However, the MiAD of metastatic RLNs was significantly larger than that of benign RLNs (Table 1).

**Table 1:** Patient characteristics of benign and metastatic RLNs

Characteristic	Benign RLNs	Metastatic RLNs	*p*-value
Sex			0.122
Male	12	31	
Female	11	14	
Age (years)	53.5 ± 10.6	56.4 ± 13.2	0.341
Maximum axial diameter			
≥ 6 mm, < 10 mm	6	20	0.913
≥ 10 mm, < 20 mm	17	24	
≥ 20 mm	0	1	
Minimal axial diameter			0.005
≥ 6 mm, < 8 mm	13	21	
≥ 8 mm, < 10 mm	9	8	
≥ 10 mm	1	16	
Signal homogeneity			0.673
Homogeneous	16	29	
Heterogeneous	7	16	
Border			0.551
Well defined	20	35	
Ill-defined	3	9	

*RLNs* Retropharyngeal lymph nodes

### Repeatability of diffusion parameters and lymph node size

The intraclass correlation coefficient values for the DWI parameters ranged between 0.835 and 0.959, those for lymph node size ranged between 0.918 and 0.985, indicating good intra- and interobserver agreement for all quantitative parameters (Table 2).

**Table 2:** Reproducibility of DWI parameters and lymph node size

Parameters	Intraobserver (95% CI)	Interobserver (95% CI)
ADC (× 10^-3^ mm^2^/s)	0.896 (0.831–0.936)	0.908 (0.851–0.943)
DDC_SEM_ (× 10^-3^ mm^2^/s)	0.935 (0.894–0.960)	0.940 (0.903–0.963)
α_SEM_	0.859 (0.771–0.913)	0.898 (0.834–0.937)
D_FROC_ (× 10^-3^ mm^2^/s)	0.944 (0.910–0.966)	0.941 (0.905–0.964)
β_FROC_	0.884 (0.811–0.928)	0.909 (0.852–0.944)
μ_FROC_ (μm)	0.923 (0.875–0.953)	0.909 (0.853–0.944)
D_CTRW_ (× 10^-3^ mm^2^/s)	0.953 (0.924–0.971)	0.949 (0.917–0.968)
α_CTRW_	0.947 (0.914–0.967)	0.952 (0.922–0.970)
β_CTRW_	0.959 (0.934–0.975)	0.950 (0.918–0.969)
Maximum axial diameter	0.918 (0.871–0.949)	0.969 (0.950–0.981)
MiAD	0.985 (0.976–0.991)	0.973 (0.957–0.983)

*ADC* Apparent diffusion coefficient, *CI* Confidence interval, *CTRW* Continuous-time random walk, *DWI* Diffusion-weighted imaging, *FROC* Fractional-order calculus, *MiAD* Minimal axial diameter, *SEM* Stretched-exponential model

### Differences in each diffusion parameter between benign and metastatic RLNs

**Fig. 4:**
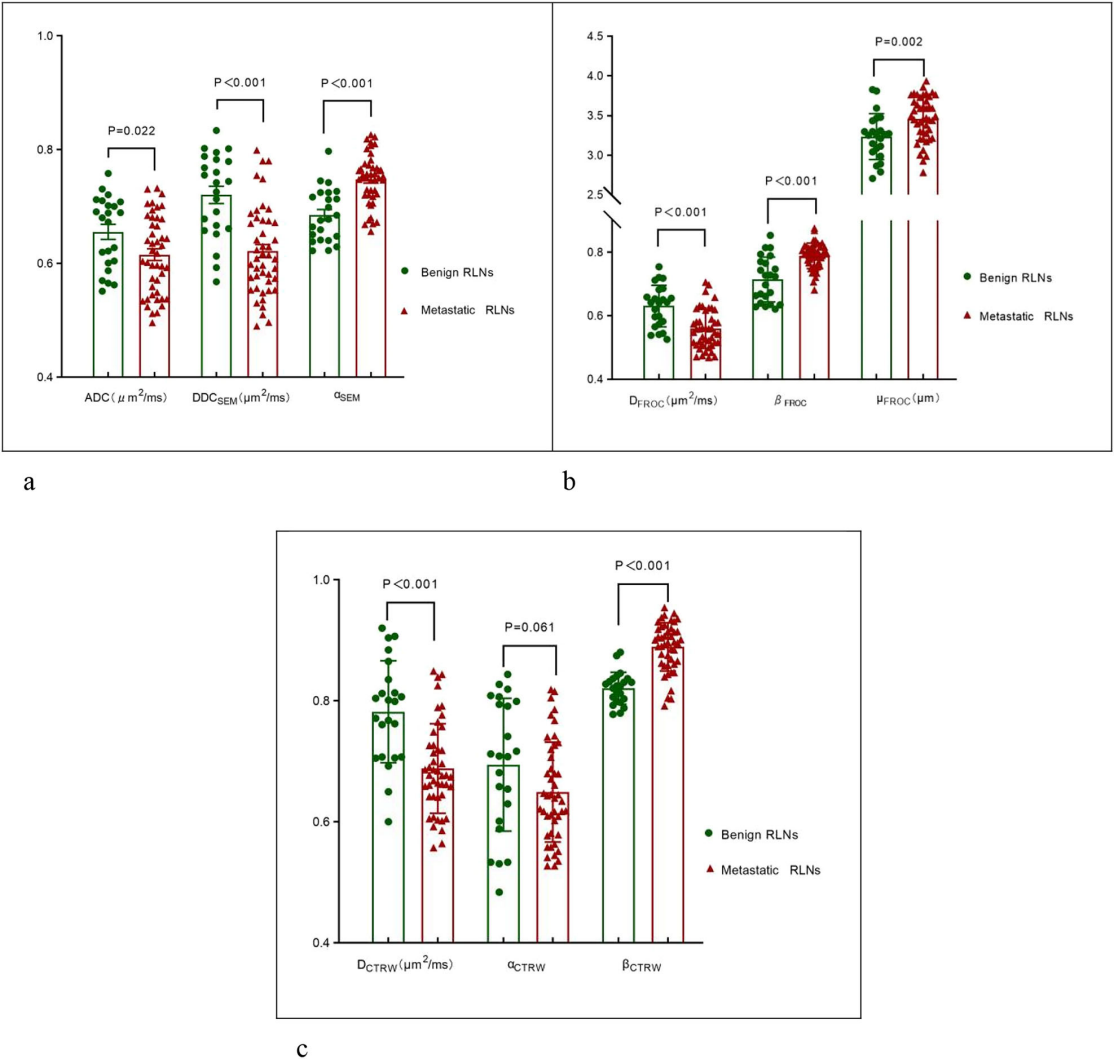
Box plots and scatter plots showing differences in quantitative diffusion parameters between benign and metastatic retropharyngeal lymph nodes. ADC, Apparent diffusion coefficient; CTRW, Continuous-time random walk; FROC, Fractional-order calculus; SEM, Stretched-exponential model. **a** Differences between parameters obtained from conventional DWI and the stretched-exponential model (SEM). **b** Differences between parameters obtained from the fractional-order calculus (FROC) model. **c** Differences between parameters obtained from the continuous-time random walk (CTRW) model.

Except for α_CTRW_, all eight diffusion parameters differed significantly between benign and metastatic RLNs. Benign RLNs exhibited higher ADC, DDC_SEM_, D_FROC_, and D_CTRW_ values, whereas metastatic RLNs had higher α_SEM_, β_FROC_, μ_FROC_, and β_CTRW_ values (Fig. 4; Table 3).

**Table 3:** Comparisons of DWI parameters between benign and metastatic RLNs

Parameters	Benign RLNs	Metastatic RLNs	Z/T value	*p-*value
ADC (μm^2^/ms)	0.655 ± 0.063	0.615 ± 0.068	2.343	0.022^a^
DDC_SEM_ (μm^2^/ms)	0.721 ± 0.073	0.622 ± 0.077	5.077	< 0.001^a^
α_SEM_	0.685 ± 0.047	0.748 ± 0.044	-5.456	< 0.001^a^
D_FROC_ (μm^2^/ms)	0.631 ± 0.065	0.559 ± 0.061	4.473	< 0.001^a^
β_FROC_	0.714 ± 0.071	0.789 ± 0.040	-4.155	< 0.001^b^
μ_FROC_ (μm)	3.235 ± 0.291	3.461 ± 0.278	-3.148	0.002^a^
D_CTRW_ (μm^2^/ms)	0.782 ± 0.084	0.688 ± 0.074	4.709	< 0.001^a^
α_CTRW_	0.694 ± 0.110	0.649 ± 0.082	1.908	0.061^a^
β_CTRW_	0.821 ± 0.027	0.889 ± 0.040	-5.542	< 0.001^b^

*ADC* Apparent diffusion coefficient, *CTRW* Continuous-time random walk model, *DWI* Diffusion-weighted imaging, *FROC* Fractional-order calculus model, *RLNs* Retropharyngeal lymph nodes, *SEM* Stretched-exponential model

^a^ Independent sample *t*-tests

^b^ Mann–Whitney U test

### Diagnostic performances of diffusion parameters and MiAD in benign and metastatic RLNs

Univariate binary logistic regression analysis identified DDC_SEM_, α_SEM_, D_FROC_, β_FROC_, μ_FROC_, D_CTRW_, and β_CTRW_ as significant predictors for distinguishing benign from metastatic RLNs. Receiver operating characteristic curve analysis showed that β_CTRW_ exhibited the highest diagnostic efficacy, with an AUC of 0.913, cutoff value of 0.830, sensitivity of 86.7%, and specificity of 91.3%. In contrast, ADC demonstrated the lowest diagnostic efficacy (AUC = 0.671, cutoff value = 0.686 μm^2^/ms, sensitivity = 82.2%, specificity of 47.8%). The MiAD yielded an AUC of 0.727, cutoff value of 9.3 mm, with 84.4% sensitivity and 52.2% specificity. The DeLong test revealed significant differences between β_CTRW_ and ADC, μ_FROC_, and MiAD (*p* = 0.002, 0.008, 0.013), as well as between DDC_SEM_, D_FROC_, D_CTRW_, and ADC (*p* = 0.002, 0.002, 0.014).

**Fig. 5:**
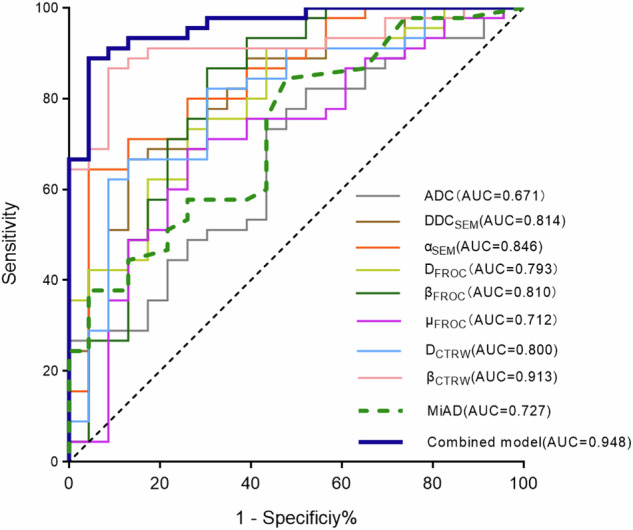
Receiver operating characteristic curves of quantitative parameters for differentiating benign and metastatic RLNs. The combined model includes β_CTRW_ and the MiAD of lymph nodes. ADC, Apparent diffusion coefficient; CTRW, Continuous-time random walk; FROC, Fractional-order calculus; MiAD, Minimal axial diameter; RLNs, Retropharyngeal lymph nodes; SEM, Stretched-exponential model

Multivariate logistic regression analysis identified β_CTRW_ as the most effective single integrated indicator. Combining β_CTRW_ with MiAD further improved diagnostic performance (AUC = 0.948, sensitivity = 91.1%, specificity = 87.0%). This combination model showed significant AUC differences compared with DDC_SEM_, α_SEM_, D_FROC_, β_FROC_, μ_FROC_, D_CTRW_, ADC, and MiAD (*p* = 0.021, 0.044, 0.011, 0.013, 0.001, 0.022, < 0.001, < 0.001), but not with β_CTRW_ alone (*p* = 0.165; Tables 4 and 5; Fig. 5).

**Table 4:** Diagnostic performance of DWI parameters and MiAD for the differentiation of benign and metastatic RLNm

Parameters	AUC	Cutoff value	Youden index	Sensitivity	Specificity
ADC (μm^2^/ms)	0.671	0.686	0.301	0.822	0.478
DDC_SEM_ (μm^2^/ms)	0.814	0.647	0.536	0.667	0.870
α_SEM_	0.846	0.746	0.601	0.644	0.957
D_FROC_ (μm^2^/ms)	0.793	0.636	0.476	0.911	0.565
β_FROC_	0.810	0.748	0.562	0.867	0.696
μ_FROC_ (μm)	0.712	3.316	0.428	0.689	0.739
D_CTRW_ (μm^2^/ms)	0.800	0.703	0.536	0.667	0.870
β_CTRW_	0.913	0.830	0.780	0.867	0.913
MiAD (mm)	0.727	9.3	0.366	0.844	0.522
Combined model	0.948	0.656	0.781	0.911	0.870

*ADC* Apparent diffusion coefficient, *AUC* Area under the curve, *CTRW* Continuous-time random walk model, *DWI* Diffusion-weighted imaging, *FROC* Fractional-order calculus model, *MiAD* Minimal axial diameter, *RLNs* Retropharyngeal lymph nodes, *SEM* Stretched-exponential model

**Table 5:** Logistic regression analyses of diffusion parameters for differentiating benign and metastatic RLNs

Variables	Univariable logistic regression	Multivariable logistic regression
	Odds ratio [95% CI] *p-*value	Odds ratio [95% CI] *p-*value
ADC (μm^2^/ms)	0.991 [0.983–0.999] 0.036	NA NA
DDC_SEM_ (μm^2^/ms)	0.983 [0.975–0.992] < 0.001	NA NA
α_SEM_	1.029 [1.015–1.044] < 0.001	NA NA
D_FROC_ (μm^2^/ms)	0.983 [0.974–0.992] < 0.001	NA NA
β_FROC_	1.026 [1.013–1.039] < 0.001	NA NA
μ_FROC_ (μm)	1.003 [1.001–1.005] 0.006	NA NA
D_CTRW_ (μm^2^/ms)	0.986 [0.978–0.993] < 0.001	NA NA
β_CTRW_	1.050 [1.027–1.074] < 0.001	1.050 [1.027–1.074] < 0.001

*ADC* Apparent diffusion coefficient, *CTRW* Continuous-time random walk model, *CI* Confidence interval, *FROC* Fractional-order calculus model, *NA* Not applicable, *RLNs* Retropharyngeal lymph nodes, *SEM* Stretched-exponential model

## Discussion

This study evaluated the clinical value of four diffusion model parameters and lymph node morphological characteristics in distinguishing between benign and metastatic RLNs. The results indicate that some non-Gaussian diffusion parameters, particularly β_CTRW_, outperform conventional ADC and MiAD in differentiating benign from metastatic RLNs. Moreover, the combined model of β_CTRW_ and MiAD demonstrates a higher AUC, contributing to improved diagnostic efficiency.

This study found that metastatic RLNs exhibited lower ADC values than benign hyperplastic lymph nodes, suggesting increased cell density in metastatic lymph nodes. This finding aligns with the results of Wang et al [18–20]. However, So et al [21] reported lower ADC values in benign RLNs, possibly due to variations in ROI delineation, *b*-value selection, and scanning protocols [40–42]. Furthermore, single-shot echo-planar imaging can introduce geometric distortions and artifacts, potentially affecting data stability.

Unlike previous studies on RLNs [17–21], this study simultaneously incorporated three non-Gaussian models (CTRW, FROC, and SEM) to capture differences between benign and metastatic RLNs using multiple diffusion parameters that reflect tissue heterogeneity. Furthermore, we compared the diagnostic efficacy of these models against ADC values and incorporated morphological features for a comprehensive analysis. To enhance data stability, we utilized the “Readout Segmentation of Long Variable Echo-trains”—RESOLVE sequence for image acquisition, thereby reducing distortions and artifacts in DWI images [43]. Previous studies have confirmed the utility of these models in lesion staging, identification, and efficacy assessment [22–38]; however, their application to RLNs remains unexplored.

DDC_SEM_ reflects the average diffusion rate within a voxel and represents a weighted sum of ADC values from multiple exponential decay components, exhibiting a strong positive correlation with ADC [23]. In this study, the correlation coefficient between DDC_SEM_ and ADC was 0.797 (*p* < 0.001), which is consistent with previous findings. D_FROC_, derived from fitting multiple *b*-values below 1,000 s/mm², functions similarly to diffusion coefficients in other diffusion models. It reflects tumor cell density, cell membrane integrity, and other structural properties influencing water diffusion in tissues, making it a more physiologically relevant indicator of true tissue diffusion [25–30]. D_CTRW_, similar to ADC, measures tissue cell density [31, 32]. However, unlike ADC, D_CTRW_ accounts for non-Gaussian diffusion behavior [33–38]. Compared with benign RLNs, metastatic RLNs exhibit increased cell density and abnormal proliferation, leading to more pronounced restricted water diffusion and lower values for these parameters. These findings are consistent with previous studies of prostate, breast and clear cell carcinoma of the kidney [27–29, 32, 33, 38].

μ_FROC_ is a measure of the average free diffusion length and is inversely correlated with tissue cellularity [28, 29]. In malignant tumors, abnormal cell proliferation restricts water molecule diffusion, leading to higher μ_FROC_ values. Previous studies have demonstrated that malignant breast lesions and high-grade bladder tumors exhibit higher μ_FROC_ values. In this study, the μ_FROC_ value of metastatic lymph nodes was lower, aligning with these earlier findings [28, 29].

α_SEM_ measures the deviation of signal decay characteristics within a voxel from the single-exponential model, with lower α_SEM_ values indicating greater inhomogeneity in exponential decay [22, 23]. In this study, benign RLNs exhibited relatively low α_SEM_ values, similar to the findings of Seo [24], who reported lower α_SEM_ values in hepatic hemangiomas compared to hepatic metastases. β_FROC_ quantifies tissue homogeneity, reflecting increased heterogeneity within a voxel [25–30]. Previous studies have demonstrated that malignant lesions typically exhibit lower β_FROC_ values [28, 29]. In this study, benign lymph nodes undergoing reactive hyperplasia showed increased proliferation of cells, such as lymphocytes, macrophages, and plasma cells, in response to viral or other stimuli [44, 45]. This resulted in increased inhomogeneity and heterogeneity in exponential decay within voxels, leading to lower α_SEM_ and β_FROC_ values.

The parameters α_CTRW_ and β_CTRW_ are novel indicators reflecting temporal and spatial diffusion heterogeneity, respectively. In a homogeneous medium, both values approach 1.00, whereas tissue heterogeneity leads to their reduction [33–35]. A lower α_CTRW_ indicates that water molecules diffuse through a more temporally heterogeneous environment, whereas an increased β_CTRW_ suggests a more spatially homogeneous environment [33–38]. In this study, both benign and malignant RLNs exhibited reduced α_CTRW_ and β_CTRW_ values. However, a significant difference was observed in only β_CTRW_ values between the two groups. This discrepancy arises because different diffusion parameters reflect distinct aspects of tissue heterogeneity. Stimulation by factors such as inflammation and viral infections induced changes in the pathological microcirculation of RLNs, altering the diffusion of water molecules within them. These alterations were more pronounced in spatial rather than temporal diffusion heterogeneity. Similar dissociations between α_CTRW_ and β_CTRW_ have been reported in previous studies exploring lesions using the CTRW model. For instance, in a study comparing patients with Parkinson’s disease and healthy controls [34], only β_CTRW_ exhibited a significant difference, while α_CTRW_ values remained comparable between the two groups. Conversely, a study differentiating prostate cancer from chronic prostatitis revealed significant differences in α_CTRW_ but not in β_CTRW_ values [35]. These findings suggest that α_CTRW_ and β_CTRW_ can characterize different types of changes in water molecule diffusion within lesions. Additionally, β_CTRW_ has demonstrated significant advantages in characterizing tissues with complex structures. For instance, in cervical cancer, β_CTRW_ exhibited superior diagnostic performance in differentiating squamous cell carcinoma from adenocarcinoma, with an AUC value of 0.836, compared to 0.664 for DDC_SEM_ and 0.642 for μ_FROC_ [37]. Furthermore, in a study predicting the Ki-67 proliferation index in cervical cancer, β_CTRW_ was identified as independent predictor, significantly enhancing the accuracy of the combined prediction model [38].

Morphological analysis of RLNs in this study showed that the MiAD was the only parameter significantly different between benign and metastatic lymph nodes, consistent with findings reported by Wang et al [20]. The diagnostic efficacy of the MiAD for distinguishing between benign and metastatic RLNs was 0.727, with a cutoff value of 9.3 mm, sensitivity of 84.4%, and specificity of 52.2%, respectively. Previous studies have also supported the utility of the MiAD in evaluating RLN metastasis [14, 15]. The current AJCC staging system lacks an optimal threshold for identifying metastatic RLNs [6]. Research has shown that a MiAD of 6.0 mm may be more appropriate than 5.0 mm [14, 15]. However, these size criteria are based on a normal population without infections or malignant lesions in the head or neck and, therefore, may not effectively differentiate benign reactive hyperplastic RLNs from metastatic lymph nodes [17–19].

Further analysis using receiver operating characteristic curves revealed that among the individual diffusion parameters, β_CTRW_ exhibited the highest diagnostic efficiency, with an AUC of 0.913, significantly outperforming ADC. This indicates that non-Gaussian parameters are more effective in reflecting tissue heterogeneity. Multivariate regression analysis confirmed β_CTRW_ as the optimal single diffusion indicator for differentiation. Combining β_CTRW_ with MiAD increased the AUC to 0.948, showing a significant improvement over all diffusion parameters except β_CTRW_ alone. These findings highlight the potential of advanced non-Gaussian diffusion models, when combined with morphological features, to provide a more comprehensive assessment of RLN heterogeneity and improve differentiation between benign and metastatic nodes, offering valuable clinical insights for treatment planning.

This study has some limitations. First, as a single-center, small cohort study, although this study initially validated the diagnostic value of diffusion parameters, further studies need to be validated by multicenter studies with large samples to confirm the efficacy of diffusion parameters in differentiating lymph nodes. Second, due to challenges in obtaining biopsies or resections of RLNs, histopathological data were unavailable. In this study, benign RLNs were identified based on MRI and endoscopic examinations in patients without nasopharyngeal or other head and neck cancers, with at least 1 year of clinical follow-up. To minimize false positives and negatives, both benign and metastatic lymph nodes were included based on a MiAD ≥ 6.0 mm. Third, diffusion parameters were measured using a two-dimensional ROI. A three-dimensional volume of interest (VOI) outlining the tissues may provide a more comprehensive assessment of heterogeneity. However, VOI delineation is labor-intensive and time-consuming, though artificial intelligence may offer a potential solution. Finally, although the current study preliminarily confirmed the value of non-Gaussian diffusion parameters in differentiating between benign and metastatic RLNs, the underlying biophysical mechanisms remain to be systematically elucidated. Therefore, future studies should focus on exploring the correlation mechanism between microenvironmental heterogeneity and diffusion patterns. This can be achieved using histopathology-radiomics registration technology to elucidate the relationship between tissue microstructure and diffusion heterogeneity coefficients. Additionally, it is necessary to quantify the impact weights of factors such as tumor cell proportion, microvessel density, and extracellular matrix on diffusion heterogeneity parameters. This will facilitate the transformation of imaging biomarkers into tools for individualized treatment decision-making.

In summary, non-Gaussian diffusion parameters derived from the CTRW, FROC, and SEM models play a significant role in distinguishing benign from metastatic RLNs, with β_CTRW_ being the most discriminative diffusion parameter. The combined application of β_CTRW_ and MiAD can further enhance diagnostic accuracy and holds promise as a noninvasive imaging biomarker for RLNs evaluation. This approach may facilitate the development of more precise clinical treatment strategies, reduce unnecessary or inappropriate radiotherapy, and provide essential imaging support for individualized treatment and prognostic assessment in NPC.

## Data Availability

The data that support the findings of this study are available from YQ. Restrictions apply to the availability of these data, which were used under license for the current study, and therefore are not publicly available. Data are, however, available from the authors upon reasonable request and with permission from YQ.

## Abbreviations

ADC: Apparent diffusion coefficient
AUC: Area under the receiver operating characteristic curve
CTRW: Continuous time random walk model
DWI: Diffusion weighted imaging
EBV: Epstein-Barr virus
FROC: Fractional order calculus model
MiAD: Minimal axial diameter
MRI: Magnetic resonance imaging
NPC: Nasopharyngeal carcinoma
RLNs: Retropharyngeal lymph nodes
ROI: Region of interest
SEM: Stretched exponential model
